# P-843. Enhancing Bacteremia Mortality Predictions: A Comparative Study of Machine Learning Models Across Databases

**DOI:** 10.1093/ofid/ofae631.1035

**Published:** 2025-01-29

**Authors:** James Kurian, Max W Adelman, Cesar A Arias, Stefano Casarin, Ashton Connor, German Contreras, Masayuki Nigo

**Affiliations:** Houston Methodist, Houston, Texas; Houston Methodist Hospital, Houston, Texas; Houston Methodist and Weill Cornell Medical College, Houston, TX; Houston Methodist Hospital, Houston, Texas; Houston Methodist Hospital, Houston, Texas; University of Texas Medical Branch, Houston, Texas; Houston Methodist Hospital, Houston, Texas

## Abstract

**Background:**

Bacteremia is a major cause of morbidity and mortality in hospitals, and multiple scoring mechanisms have been developed to help with risk assessment. In the setting of bacteremia, the Pitt bacteremia score (PBS) has been used. This study develops ML models to predict mortality in bacteremic patients, comparing their performance against the PBS.

Characteristics of Patients

**Figure:**
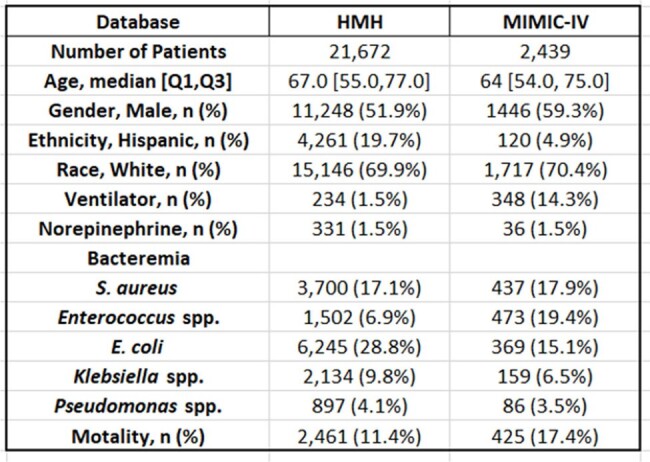

ECI: Elixhauser Comorbidity Index

**Methods:**

We retrospectively identified patients aged over 18 years who developed bacteremia in the Houston Methodist Hospital System (HMH) from June 2016 to June 2023. True bacteremia was defined based on CDC definitions. MIMIC-IV data, a comprehensive, de-identified electronic health record (EHR) database of critical care patients from Boston, USA, served as an external validation dataset. We recorded maximally perturbed values of 34 clinical and laboratory variables within 48 hours before the first bacteremia. Missing values were imputed using multiple imputation chain equation (MICE) method after preprocessing datasets. Each dataset was split into 70:30 training and test datasets. Models including PBS, Logistic Regression (LR), Random Forest (RF), LightGBM (LGBM), and XGBoost (XGB), along with hyperparameter tuning via Optuna, were used in classification tasks to predict 30-day all-cause mortality. AUROCs (Area Under the Receiver Operating Characteristics) for each model on test dataset were created, and they were statistically compared with DeLong’s tests. Probability cutoffs for each model were decided to maintain a specificity close to 95%.

Model Performance of ML Models and Pitts Score against Mortality

**Figure:**
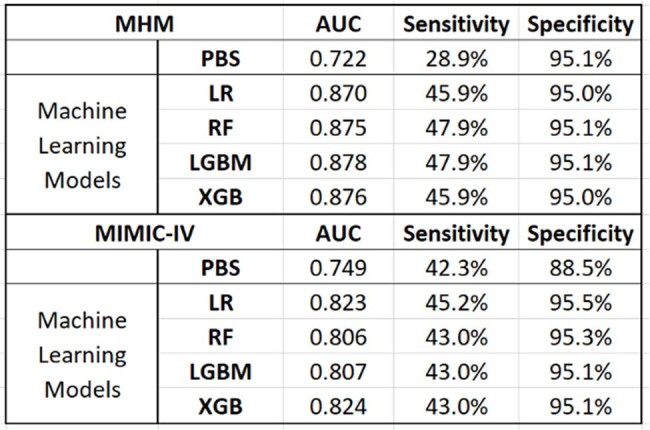

**Results:**

Across HMH and MIMIC-IV, 21,972 and 2,439 patients experienced BSIs, respectively. Table 1 summarizes characteristics of the patients. *E. coli* and *Enterococcus* spp. were the most common pathogens in HMH and MIMIC-IV, respectively. *S. aureus* made up 17% of bacteremia. All the ML models exhibited higher AUROCs than that of the PBS. LGBM and XGB achieved the highest AUROCs of 0.878 and 0.824, respectively, surpassing PBS (p < 0.05).

**Conclusion:**

ML models demonstrated higher AUROC and superior performance than that of PBS. ML allows for vast quantities of input data. This ability to process and integrate diverse features may contribute to personalized medicine in infectious diseases.

**Disclosures:**

**All Authors**: No reported disclosures

